# Using Pressure-Driven Membrane Processes to Remove Emerging Pollutants from Aqueous Solutions

**DOI:** 10.3390/ijerph18084036

**Published:** 2021-04-12

**Authors:** Asunción María Hidalgo, Gerardo León, María Dolores Murcia, María Gómez, Elisa Gómez, José Luis Gómez

**Affiliations:** 1 Chemical Engineering Department, Campus de Espinardo, University of Murcia, 30100 Murcia, Spain; md.murcia@um.es (M.D.M.); maria.gomez@um.es (M.G.); egomez@um.es (E.G.); carrasco@um.es (J.L.G.); 2 Chemical Engineering and Environmental Department, University of Cartagena, 30203 Cartagena, Spain; gerardo.leon@upct.es

**Keywords:** emerging pollutants, nanofiltration, reverse osmosis, solution-diffusion model

## Abstract

Currently, there is great concern about global water pollution. Wastewater generally contains substances called emerging pollutants, and if the removal of these pollutants is not given sufficient attention, the pollutants can enter into the water cycle and reach the water supply for domestic use, causing adverse effects on the well-being of people. In order to avoid this menace, a multitude of techniques to reduce the high concentration levels of these substances dissolved in water are being researched and developed. One of the most-used techniques for this goal is the physical-chemical separation of contaminants in water through membrane technology. In this study, different membranes were tested with the objective of investigating the removal of three emerging pollutants: caffeine, metformin, and methyl-paraben. Initially, a nanofiltration (NF) membrane was selected, and the influence of pressure was evaluated in the rejection coefficients and permeate fluxes. Next, a screening of three new membranes to remove methyl paraben was completed. The influence of the operating variables, working pressure, and methyl paraben-feed concentration was checked. Finally, the solution-diffusion model was applied to predict the behavior of the different membranes in the removal of methyl paraben. A good correlation between experimental and calculated values of permeate flux and methyl paraben concentration was obtained.

## 1. Introduction

Three quarters of the total industrial discharge, which contains highly-polluting substances, is released into the sea without any prior treatment. Among these substances, there are compounds that are difficult to degrade due to their complex structure, which makes them stable and, consequently, poorly biodegradable. These compounds are called emerging pollutants [1]. 

An emerging pollutant or micro-pollutant is a substance whose presence in water poses a danger to human health and the environment, since it accumulates in waterways, seas, and aquifers, causing the loss of aquatic biosystems [2]. The most common emerging pollutants are usually classified into the following groups: drugs, perfluorinated compounds, hormones, drugs of abuse, and personal care and hygiene products [3].

One of the main problems caused by these compounds is that once they enter the water cycle, they are very difficult to remove. Therefore, techniques continue to be developed to remove these compounds in wastewater treatment plants, and thus minimize their concentration in the water that is discharged into the aquatic environment. Biological, chemical, and physical treatments have been described in the literature, each presenting some advantages and disadvantages [4,5,6,7,8,9,10,11,12,13]. Nevertheless, membrane removal technology is well known for its efficiency, low cost, and easy operation.

To date, the studies carried out have tried to explain the process of elimination of some families of emerging pollutants in water [14,15]. One of the most studied contaminants is caffeine. Wang et al. studied the behavior of the DESAL HL nanofiltration membrane for the removal of 40 trace organic compounds, including caffeine. According to the authors, the steric hindrance effect was the primary mechanism that contributed to the rejection of the trace organic compounds by the nanofiltration membrane [16]. In addition, the rejection rate for methyl paraben decreased when the water flux increased. Mahlangu et al. used a polyamide Dow-Filmtec NF-270 membrane to remove caffeine from a water solution, obtaining a rejection coefficient of 84% [17].

Very few studies have been carried out using membranes for the elimination of metformin from wastewater. In a test carried out by Foureaux et al., an attempt was made to separate four drugs present in the Rio Doce using nanofiltration (NF) and reverse osmosis (RO) membranes. One of the drugs was metformin. These drugs had similar volumes and molecular weights, but the dissociation and hydrophobicity constants, which are important in membrane separation, were different. The results obtained for metformin showed an elimination coefficient close to 94%, which reinforces the idea that membrane separation is the optimal technology [18].

However, in the literature there are references to other drugs and their elimination through membranes. For example, Acero et al. [19] combined ultrafiltration membranes with different micelles to optimize the rejection coefficient of ultrafiltration membranes for different drugs. The cetyltrimethylammonium bromide membranes were found to be the most effective, increasing the rejection coefficient to 95%. This method with micelles turns out to be very efficient but also involves a high economic cost.

Al-Rifai et al. [20] conducted a test with microfiltration and reverse osmosis membranes to reduce the concentration of 11 drugs present in wastewater. Excellent results were obtained, as the presence of drugs in the post-treatment wastewater were reduced by at least 97%.

Lopez-Muñoz et al. [21] used NF-90 and NF-270 membranes for the elimination of various emerging pollutants present in wastewater. They concluded that the rejection coefficient depends on the physicochemical properties of the solute and on the working pressure. 

The application of pressure-driven membrane processes for the removal of low molecular weight organic compounds from aqueous solutions has been described in several recent publications, which discussed factors such as particle size, form, charge, and octanol-water coefficient [22,23,24,25]. It is clear that the removal efficiency depends on the membrane type and solute, and the interaction between them. Temperature, pH, pressure, and concentration also influence rejection [26,27].

Whether nanofiltration should be used in the treatment of wastewater containing emerging pollutants depends on the rejection capacity of the membranes and the permeate flux [15,28,29,30,31]. More recently, new membrane materials for this application have been developed [32].

In the present work, caffeine (C), metformin (MF), and methyl paraben (MP) were selected as examples of emerging contaminants to be treated using a polyamide nanofiltration membrane. The influence of different operating pressures on the permeate flows and rejection percentages was studied with regard to the elimination efficiency for the three pollutants.

The selection of the three pollutants was based on their wide commercial use and the existence of differences in some of their physicochemical properties such as structure, size, molecular weight, polarity, and acid-base strength. All three studied pollutants have a molecular weight <200 g/mol.

In particular, the methyl paraben pollutant was treated with different nanofiltration and reverse osmosis membranes, and an initial characterization of these membranes was carried out in order to obtain their permeability to the solvent (distilled water) and their behavior towards saline solutions by observing the rejection coefficients and the membrane permeate values. Afterwards, the influence of operating variables such as working pressure and methyl paraben concentration in the feed was checked. 

Finally, the solution-diffusion model was applied to fit the experimental data corresponding to the methyl paraben assays. 

## 2. Materials and Methods

### 2.1. Materials

- Caffeine, C_8_H_10_N_4_O_2_, molecular weight 194.9 g/mol, purchased from Sigma-Aldrich Inc.
- Metformin, C_4_H_11_N_5_, molecular weight 129.16 g/mol, purchased from Sigma-Aldrich Inc.
- Methyl paraben, C_8_H_8_O_3_, molecular weight 152.15 g/mol, purchased from Alfa Aesar.
- Sodium chloride, NaCl, molecular weight is 58.4 g/mol, supplied by Panreac.
- Hydrous magnesium chloride, MgCl_2_ 6H_2_O, molecular weight is 203.30 g/mol, supplied by Panreac.

The membranes were provided by Alfa Laval and manufactured by Dow Chemical. The characteristics of the membranes are described in Table 1.

### 2.2. Experimental Equipment

**Figure 1:**
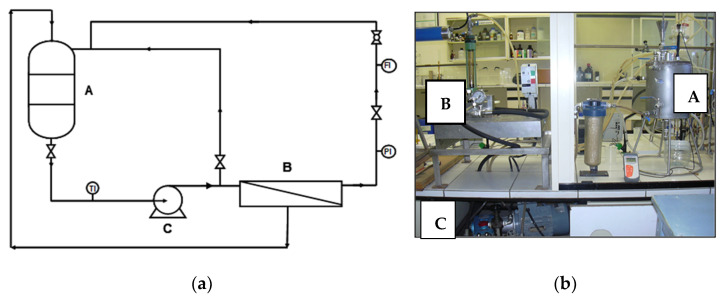
(**a**) Scheme of the membrane test module. (**b**) Picture of the membrane test module.

All the assays were performed in an INDEVEN flat membrane test module, which is designed for a maximum operating pressure of 70 × 10^5^ Pa and provides data concerning the behavior of the membranes in cross flow conditions with a reduced surface area (3 × 10^–3^ m^2^), low feed flow, and short times. Figure 1 shows a diagram of the experimental unit. The feed tank (**A**) is a closed stainless steel vessel, with a capacity of 12 × 10^−3^ m^3^, equipped with a water coiling coil that allows a constant feed temperature. The membrane module (**B**) supports the membrane. The feed solution is fed through the membrane module by means of a high pressure pump (**C**). Recycling of both concentrate and permeate was carried out in order to keep the feed concentrations nearly constant and so simulate a continuous process in a quasi-stationary state.

### 2.3. Methods

Samples of permeate, concentrate, and feed flow were measured by a colorimetric method using a Shimadzu UV-160 spectrophotometer. The concentrations of caffeine, metformin, and methyl paraben were analyzed at 275, 240, and 260 nm wavelengths, respectively.

Membrane performance was measured in terms of membrane rejection coefficient (% R) and permeate flux (J_p_). 

For dilute aqueous mixtures consisting of water and a solute, the selectivity of a membrane is usually expressed in terms of the solute rejection coefficient [33], which is defined as a percentage by Equation (1),

(1) $$\%\;R=\;\left(1-\frac{C_{p}}{C_{f}}\right)\cdot \;100$$

where C_p_ and C_f_ are the solute concentrations in the permeate and feed streams, respectively.

The permeate flux was calculated according to the following Equation (2),

(2) $$J_{p}=\;\frac{Q_{p}\;\cdot \rho \;}{S}$$

where Q_p_ is the permeate flow (m^3^/s), ρ the density of the solution (kg/m^3^), and S is the effective membrane area (m^2^).

### 2.4. Experimental Series

Different experimental series were carried out in order to check the behavior of the system in the removal of the mentioned emerging pollutants.

Initially, to determine the solvent permeability of different membranes, experiments at different operating pressures (10, 15, 20, and 25 bar for nanofiltration membranes and 20, 25, 30, and 35 bar for reverse osmosis membranes) were carried out.

To determine the membranes’ selectivity, aqueous solutions (MgCl_2_ and NaCl) of 1 g/L were used to carry out the experimental assays. Different operating pressures were applied in a similar mode to that of the permeability coefficient determination assays.

To study the influence of the operating pressure on the permeate flows and the rejection coefficients using the NF99 membrane, a 25 mg/L concentration of caffeine and methyl paraben and a 10 mg/L concentration for metformin were used. For the screening of different membranes in the removal of methyl paraben, a constant pollutant concentration of 25 mg/L was fixed and different pressure ranges for nanofiltration and reverse osmosis membranes were applied, between 10 and 25 bar and 20 and 35 bar, respectively.

Finally, in the study of the influence of methyl paraben feed concentration, the tests were performed at concentrations of 12.5, 25, 50, and 100 ppm for the NF99HF, RO90, and RO99 membranes.

### 2.5. Theoretical Solution-Diffusion Model

Solution-diffusion models were used to depict mass transfer through the membranes after experimentally determining the constants of the models. System mass balances, together with solution-diffusion mass transfer models, were used to simulate the system in operation [34,35,36]. The model equations have been previously discussed in other research works [37]. Other authors have applied the model to different organic compounds in nanofiltration and reverse osmosis membranes [38,39]. The equations used to predict the behavior of the system are as follows:

- To obtain the solute concentration in the permeate:
  (3) $$C_{p}=\;\frac{C_{f}}{1+\;\frac{A_{w}\cdot \Delta P}{B_{s}\cdot C_{w}}-\;\frac{\Psi \cdot C_{f}\;\cdot A_{w}}{B_{w}\cdot C_{w}}}$$
- For the permeate flux:
  (4) $$J_{p}=\;[\;\Delta P-\;\Psi \cdot {\Delta C}_{f}+\frac{C_{f}}{1+\;\frac{A_{w}\cdot \Delta P}{B_{s}\cdot C_{w}}-\;\frac{\Psi \cdot C_{f}\;\cdot A_{w}}{B_{w}\cdot C_{w}}}]$$

From Equations (3) and (4) it is possible to determine the solute concentration in the permeate and the volumetric permeate flux as a function of the solute feed concentration, C_f_, the operating pressure, P, the osmotic pressure coefficient, ψ, and the membrane size and characteristics expressed by the constants A_w_ (water permeability constant) and B_s_ (solute permeability constant). C_w_ is the solvent permeate concentration.

## 3. Results and Discussion

### 3.1. Characterization of Different Membranes

For the initial characterization of the selected membranes, tests were first carried out with distilled water to obtain the solvent permeability and then with the saline solutions of NaCl and MgCl_2_ to determine the solute selectivity of the reverse osmosis and nanofiltration membranes, respectively.

#### 3.1.1. Solvent Permeability

To determine the permeability coefficient of the solvent, the following equation was used:

(5) $$J_{w}={\;A}_{w}\cdot \left(\Delta P-\Delta \Pi \right)$$

where J_w_ is the permeate mass flow (kg/m^2^·s), A_w_ is the solvent permeability coefficient (s/m), and ΔP and ΔΠ are the hydraulic and osmotic pressure gradients, respectively.

The osmotic pressure gradient exerted by the solvent is negligible, since the solute concentration is zero, so Equation (5) can be expressed as follows:

(6) $$J_{w}={\;A}_{w}\cdot \left(\Delta P\right)$$

Subsequently, a linear least squares regression is established, representing the permeate mass flow, J_w_, against the applied hydraulic pressure gradient, ΔP, obtaining a straight line whose slope is the solvent permeability coefficient, A_w_. Table 2 shows the solvent permeability coefficients for each one of the tested membranes.

When comparing the permeability values obtained for the different membranes with those reported in the literature, it is observed that they are approximately of the same order of magnitude [27,40].

#### 3.1.2. Selectivity and Performance of Different Membranes

The characterization of reverse osmosis membranes is often carried out using sodium chloride solutions, and for nanofiltration membranes, divalent salt solutions are normally used. In this research, two salt solutions were used: sodium chloride and magnesium chloride. To determinate the selectivity of the different membranes, the rejection coefficient was established using Equation (1).

The experimental values obtained for the permeate flux and rejection coefficient were fitted to the solution-diffusion model [34]. As a result, the permeability coefficient for the solute (B_s_) for each salt solution was obtained.

Table 2 shows the B_s_ values for each salt solution assayed, which are very close to those obtained by previous authors [27,40]. 

### 3.2. Removal of Different Emerging Pollutants Using A Polyamide NF Membrane

Hydraulic pressure is one of the most important parameters in membrane operations, as it is necessary to overcome osmotic pressure. As a result of this process, a permeate flux through the membrane is obtained.

**Figure 2:**
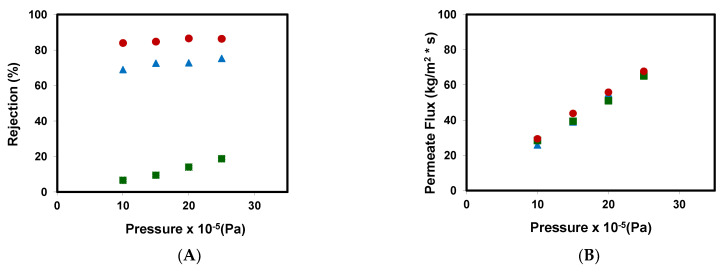
(**A**) Rejection coefficients and (**B**) permeate fluxes obtained with the NF99 membrane at different pressures for the different emerging pollutants assayed: (■) methyl paraben, (●) caffeine, and (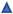) metformin.

Figure 2A represents the rejection coefficients obtained for the different emerging pollutants assayed at different pressures. Its values ranged from 80% to 90% for caffeine, from 70% to 80% for metformin, and from 5% to 20% for methylparaben, they all increased with the increase of pressure.

To discuss the behavior of the NF99 membrane on the removal of the three emerging pollutants selected, it is very important to know the physicochemical parameters of each pollutant. Table 3 shows the main physicochemical parameters of these compounds.

According to the pK_a_ values of the three chemical molecules, at the neutral pH of the experiments, both caffeine and methyl paraben are in non-charged form while metformin has a positive charge.

Different studies have described that in the case of uncharged molecules, the size and the logK_ow_ are the main parameters governing rejection in nanofiltration processes [2,14,41,42,43], with the rejection coefficient being higher at higher values of molecular size and lower values of logK_ow_. The higher caffeine rejection value compared to that of methylparaben can be explained by both its higher molecular weight 194.19 g/mol and lower logK_ow_ (−0.07); in comparison, the values of methylparaben are 152.15 g/mol and +1.96, respectively. The results obtained for the caffeine rejection coefficient are in line with those obtained by other authors [10,16,17,23,25].

The high rejection coefficient of metformin, similar to that obtained by other authors [18], can be related to the presence of a net positive charge in its molecule under the experimental conditions assayed. Charged molecules are solvated in water, which enlarges their effective size and increases their rejection [14]. The net positive charge of metformin favors its solvation in water, which leads to an increase of its molecular size. Moreover, the Stokes radius of metformin (0.318 nm) is higher than that of methylparaben (0.270 nm). Both facts make the rejection of metformin much higher than that of methylparaben, despite its lower molecular weight.

Table 4 shows a comparison of the removal of emerging pollutants between previous studies and the results obtained in this work. It can be seen in Figure 2B that an increase in hydraulic pressure causes an increase in the pressure differential, which is responsible for the passing of the feed solution through the membrane. Furthermore, the permeate flux values obtained for each pollutant are very similar for the different pressures assayed (there is a linear increase), which suggests that the influence of the type of pollutant on the membrane permeate flow is minimal in this concentration and pressure range.

The selectivity of the NF99 membrane for methyl paraben is very low, as was previously stated. A new experimental series using different membranes (NF99HF, RO90, and RO99) was carried out in order to make a comparative study.

### 3.3. Screening of Different Membranes for the Removal of Methyl Paraben

#### 3.3.1. Influence of Pressure

**Figure 3:**
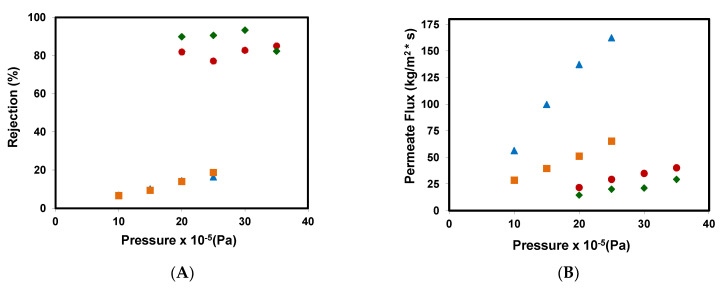
(**A**) Rejection coefficients and (**B**) permeate fluxes obtained for the removal of methyl paraben at different pressures for the different membranes assayed: (■) NF99, (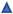) NF99HF, (♦) RO99, and (●) RO90.

A study of the influence of the operating pressure on the permeate flows and the rejection coefficients was carried out under the experimental conditions shown in the Material and Methods section. Figure 3A shows the rejection coefficients obtained from different membrane assays. The results attained for the reverse osmosis membranes show that the selectivity of both membranes was high (80% and 90% for RO90 and RO99, respectively). However, NF99 and NF99HF membranes obtained very low rejection coefficients, between 5–25%. This improved performance is related to the different molecular weight cut off values of the membranes. On the other hand, the rejection coefficient increased when going from 10 to 25 bar, but when the pressure was increased to 30 and 35 bar for the RO99 reverse osmosis membrane, a lower rejection coefficient was obtained.

In the study carried out by López-Ortiz et al. about the removal of parabens from water using a combined treatment of magnetic ion exchange resins and subsequent filtration through nanofiltration membranes, methyl paraben showed the poorest removal yield (31%) when using MIEX^®^ GOLD resin, while the best result (80%) was achieved for butylparaben. The best results were obtained in all the experimental series with the compounds that had the longer alkyl chains. According to the authors, the small molecules together with the hydrophobic character of the membrane surface makes the membrane surface control the removal yield. Compounds with higher hydrophobic character are easier to remove than those with lower hydrophobicity [12].

As it can be seen in Figure 3B that although the membranes work at different ranges of operating pressures, in general, the permeate mass flow increases as the operating pressure increases. For NF99 and NF99HF membranes, the almost linear increase with respect to pressure indicates that the fouling effect and the polarization effects are not very significant. Meanwhile, for reverse osmosis membranes, a very slight increase in flow can be observed as pressure increases.

When comparing the results obtained with those in the literature, it was found that they are similar to those referenced by other authors [12,16].

#### 3.3.2. Influence of Methyl Paraben Feed Concentration

For the study of the influence of the feed concentration of methyl paraben, the tests were performed at different concentrations according to the Material and Methods section. The NF99 membrane was eliminated in this experimental series since the behavior of this nanofiltration system was very similar to that of the NF99HF membrane. Permeate fluxes and rejection coefficients were compared for different concentrations at pressures of 20 and 25 bar. These pressure values were selected because they are coincident for all the membranes assayed.

**Figure 4:**
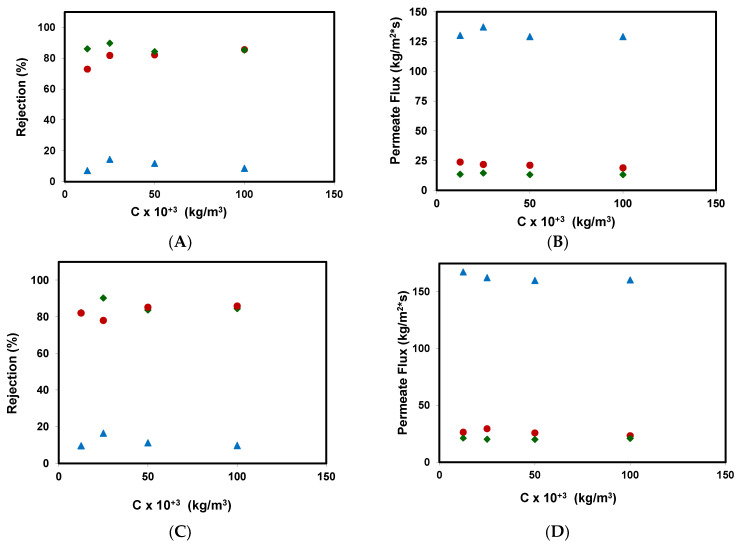
Rejection coefficients and permeate fluxes obtained for the removal of methyl paraben at different feed concentrations for the different membranes assayed. (**A**,**B**) for an applied pressure of 20 bar; (**C**,**D**) for an applied pressure of 25 bar: (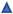)NF99HF, (♦) RO99, and (●) RO90.

Figure 4 shows the influence of methyl paraben feed concentration in the rejection coefficients and permeate fluxes obtained for the different membranes assayed: (A) and (B) for an applied pressure of 20 bar, (C) and (D) for an applied pressure of 25 bar.

The rejection coefficients obtained did not vary much with an increasing concentration of methyl paraben in the feed. A very small rejection coefficient is expected for the nanofiltration membrane, which is in a range of 5–15%. These results are similar to those obtained by Wang et al. using a DESAL HL nanofiltration membrane in the removal of methyl paraben [16]. The authors discuss that the lower rejection could be explained by methyl paraben having a moderate hydrophobic character with a logP value of 1.882, and the other compounds that were studied could interact strongly with the surface of the membrane material (H-bonding or pi-pi interactions). In the same way, reverse osmosis membranes obtained rejection coefficients of around 80%, sometimes reaching 90% for the RO99 membrane.

On the other hand, in the tests carried out with a higher concentration of the contaminant, the rejection coefficients for the RO90 membrane were very similar to those of the RO99 membrane.

As it can be seen in Figure 4B,D, there is no significant influence of the concentration of methyl paraben on the permeate fluxes, at the range of concentrations tested. As a result, no evidence of fouling phenomena was obtained.

#### 3.3.3. Fitting the Solution-Diffusion Model

**Figure 5:**
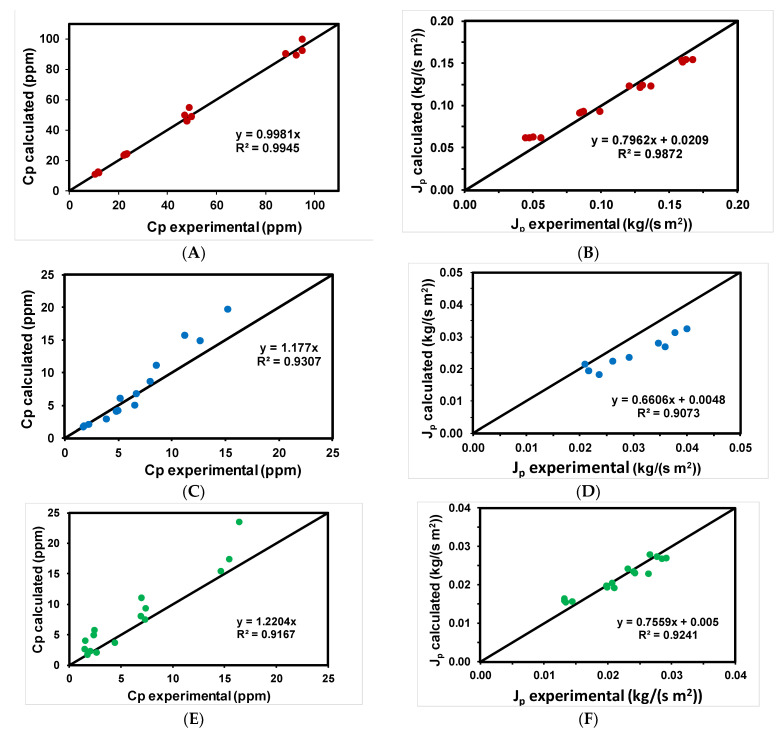
Experimental data of rejection coefficients and permeate fluxes versus those calculated with the solution-diffusion model for the different membranes tested for the removal of methyl paraben: (●) NF99HF, (●) RO90, and (●) RO99.

Table 5 shows the values of the different parameters obtained using methyl paraben as an emerging pollutant for the different membranes tested. From the estimated values of the parameters of the model (B_s_ and ψ) and using Equations (3) and (4), theoretical values of permeate flux and methyl paraben concentration were calculated and compared with the experimental values. Figure 5A–F shows that, in general, calculated and experimental concentrations of methyl paraben in the permeate and permeate flows were very close, as shown by the good approximation to the diagonal. The NF99HF membrane showed a good correlation in all cases.

## 4. Conclusions

Different nanofiltration and reverse osmosis membranes were tested for the removal of three widely used emerging pollutants: caffeine, metformin, and methylparaben. Initially, the NF99 membrane was used and though high rejection coefficients were obtained for caffeine and metformin removal in the selected range of pressures, the selectivity of the membrane towards methyl paraben was quite low, probably due to the high dipole moment of the molecule and its moderate hydrophobic character. For this reason, another nanofiltration membrane, NF99HF, and two reverse osmosis membranes, RO90 and RO99, were assayed for methyl paraben elimination.

An initial characterization of the membranes was carried out, and the values obtained for the solvent and solute permeabilities match the ones calculated in previous studies. In the screening of the different membranes for the removal of methyl paraben, when the pressure influence was checked, the NF99HF membrane showed low selectivity, as was previously shown with the NF99 membrane. Therefore, it can be concluded that NF membranes are not convenient for the treatment of methyl paraben in the working conditions assayed. In contrast, both the RO90 and the RO99 membranes presented high rejection coefficients, above 80% in all cases.

On the other hand, the linear increase of the permeate mass flow when increasing the operating pressure indicates that there were no fouling or polarization effects in the range tested. Regarding the influence of the methyl paraben feed concentration, it was almost negligible under the operating conditions assayed and, as expected, the selectivity of the NF membrane was low, while good rejection coefficients between 80 and 90% were attained with the reverse osmosis membranes, proving once again that these are the most appropriate membranes for methyl paraben removal, while the NF ones can be used for caffeine and metformin with good results.

Although RO membranes can efficiently remove almost all kinds of emerging pollutants and they are less influenced by electrostatic and hydrophobic effects in comparison to that of NF membranes, the capital and operational costs of osmosis systems should be considered carefully since RO units are operated under high pressure.

Finally, it has been proven that the solution-diffusion model can be applied to predict the system behavior in the removal of methyl paraben with the different membranes used in the present work.

New materials should be studied in future works in this field, since it is necessary to identify membranes that exhibit a higher removal efficiency together with unprecedented permeation rate.

## Figures and Tables

**Table 1:** Main characteristics of the NF and RO membranes used.

Provider	Alfa Laval	Alfa Laval	Alfa Laval	Alfa Laval
Manufacturer	Dow Chemical	Dow Chemical	Dow Chemical	Dow Chemical
Product denomination	NF99	NF99HF	RO90	RO99
Type	Thin-film composite	Thin-film composite	Thin-film composite	Thin-film composite
Composition	Polyamide	Polyamide	Polyamide	Polyamide
Molecular weight cut-off (MWCO) (Da)	≤200	≤200	-	-
Membrane surface area (m^2^)	0.003	0.003	0.003	0.003
Maximum pressure (N/m^2^)	55 × 10^5^	55 × 10^5^	55 × 10^5^	55 × 10^5^
MgSO_4_ rejection (%)	≥97	≥98	≥99	≥99
pH range	3–10	3–10	2–11	2–11
Maximum temperature (°C)	50	50	60	60

**Table 2:** Characterization of the different membranes. Experimental values for solvent permeability coefficients (A_w_) and solute permeability coefficients (B_s_).

	NF99 ^a^	NF99HF	RO90	RO99
A_w_ (s/m)	1.665 × 10^−8^	6.175 × 10^−8^	8.536 × 10^−9^	7.548 × 10^−9^
B_s_ (NaCl) (m/s)	6.705 × 10^−6^	2.777 × 10^−6^	1.135 × 10^−6^	6.551 × 10^−7^
B_s_ (MgCl_2_) (m/s)	1.632 × 10^−7^	4.496 × 10^−7^	2.022 × 10^−7^	1.231 × 10^−7^

^a^ [27].

**Table 3:** Physicochemical properties of the emerging pollutants studied.

Emerging Pollutants	Caffeine	Metformin	Methyl Paraben
Molecular weight (g/mol)	194.19	129.16	152.15
Solubility in water (25 °C) mg/L	2.16 × 10^4^	1.06 × 10^6^	2.50 × 10^3^
log K_ow_	−0.07	2.64	1.96
pK_a_	−0.13–1.22 ^f^	12.4	8.5
Topological polar surface area (Å^^2^^)	58.4	91.5	46.5
Charge	Neutral	Positive	Neutral
Dipole moment (D)	3.40–3.70 ^a, b^	0.412 ^c^	1.50
Stokes radius (nm)	0.318 ^d^	0.328 ^e^	0.270 ^d^
Chemical structure	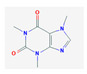	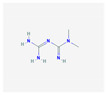	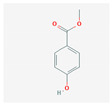

^a^ [25], ^b^ [23], ^c^ [41], ^d^ [16], ^e^ [18], ^f^ [43].

**Table 4:** Comparison of the removal of emerging pollutants between previous studies and this study in terms of water flux and rejection.

Emerging Pollutant	Membrane	Experimental Conditions	J_p_ (L/m^2^ h bar)	Rejection (%)	References
Caffeine	UF	4 bar	21.6	11.5	Acero et al., 2012. [19]
Caffeine	NF-270	7 bar	65.0	84.0–86.0	Mahlangu et al., 2014. [17]
Caffeine	NTR 7450	10–800 mg/L at 16.5 bar	-	45.4–35.0	Shirley et al., 2014. [23]
Caffeine	DESAL HL	-	-	58.0	Wang et al., 2015. [16]
Caffeine	NF90	15 bar	-	92.0	Licona et al., 2018. [25]
Caffeine	BW30	15 bar	-	92.0–95.0	Licona et al., 2018. [25]
Caffeine	NF99	25 mg/L at 10 bar	105.8	84.0	This work
Metformin	DK	35.99 ng/L at 10 bar	-	94.0	Foureaux et al., 2019. [18]
Metformin	NF99	10 mg/L at 10 bar	79.2	70.0	This work
Methyl paraben	DESAL HL	-	-	21.0	Wang et al., 2015. [16]
Methyl paraben	NF90	-	-	60.0	López-Ortíz et al., 2018. [12]
Methyl paraben	DESAL HL	-	-	62.0	López-Ortíz et al., 2018. [12]
Methyl paraben	NF99	25 mg/L at 10 bar	102.7	8.0	This work
Methyl paraben	NF99HF	25 mg/L at 10 bar	180.0	8.0	This work
Methyl paraben	RO90	25 mg/L at 20 bar	50.4	80.0	This work
Methyl paraben	RO99	25 mg/L at 20 bar	43.2	90.0	This work

**Table 5:** Solute permeability coefficients and Ψ parameters for the different membranes obtained using the solution-diffusion model in the removal of methyl paraben.

	NF99HF	RO90	RO99
B_s_ (MP) (m/s)	8.19 × 10^−4^	3.82 × 10^−6^	4.41 × 10^−6^
Ψ (MP) (m^2^/s^2^)	4.183 × 10^6^	10.28 × 10^6^	2.244 × 10^6^

## Data Availability

Data presented in this study is available within the article. There is no supplementary material.
